# Development of a new HISCL automated CXCL9 immunoassay

**DOI:** 10.1038/s41598-023-32513-8

**Published:** 2023-04-01

**Authors:** Takehiro Hasegawa, Maho Yoshida, Shunsuke Watanabe, Takami Kondo, Hideo Asada, Atsushi Nakagawa, Keisuke Tomii, Masami Kameda, Mitsuo Otsuka, Koji Kuronuma, Hirofumi Chiba, Shinji Katayanagi, Yasunari Miyazaki, Akio Mori

**Affiliations:** 1 Research and Development Division, Sysmex R&D Centre Europe GmbH, Falkenried 88, 20251 Hamburg, Germany; 2 Scientific Affairs, Sysmex Corporation, 1-3-2, Murotani, Nishi-Ku, Kobe, Hyogo 651-14 2241 Japan; 3 Central Research Laboratories, Sysmex Corporation, 4-4-4, Takatsuka-Dai, Nish Ward, Kobe, Japan; 4 Department of Dermatology, Nara Medical University School of Medicine, 840 Shijo, Kashihara, Nara 634-8522 Japan; 5 Kobe City Medical Centre General Hospital, 2-1-1, Minamimachi, Minatojima, Chuo Ward, Kobe, Japan; 6 Department of Respiratory Medicine and Allergology, Sapporo Medical University School of Medicine, Sapporo, Hokkaido Japan; 7 Department of Respiratory Medicine, Tokyo Medical and Dental University, 1-5-45 10 Yushima, Bunkyo-Ku, Tokyo, 113-8519 Japan; 8 National Hospital Organization, Sagamihara National Hospital, Clinical Research Centre, Sagamihara, Japan

**Keywords:** Medical research, Biomarkers, Immunology, Cytokines

## Abstract

C–X–C motif chemokine ligand 9 (CXCL9), a candidate biomarker, reflects type 1 (T1) inflammation pathology. Here, we report the analytical performance and clinical characteristics of a new CXCL9 reagent for a fully automated immunoassay device. We evaluated the limits of blank, detection, and quantitation (LoQ) along with other efficacy parameters, and the ability of the assay to report patient health, COVID-19 status, and the presence of asthma and/or interstitial lung diseases (ILDs). The coefficient of variation for 5-day total precision using two instruments was 7% across two controls, serum, and plasma panels. LoQ of 2.2 pg/mL suggested the efficacy of the assay in detecting T1 inflammation in plasma or serum; no cross-reactivity or interference was observed. We identified high serum CXCL9 levels in samples from patients with acute COVID-19 infections (*n* = 57), chronic bird-related hypersensitivity pneumonitis (*n* = 61), asthma (*n* = 194), and ILDs (*n* = 84) compared to healthy individuals (< 39.0 pg/mL). Furthermore, CXCL9 levels increased with age in asthma patients, and an opposite trend was observed for T2 inflammatory factors. These results suggest the utility of the automated CXCL9 immunoassay for measuring CXCL9 in clinical samples and reflect its role in T1 inflammation.

## Introduction

C–X–C motif chemokine ligand 9 (CXCL9) is a 103-amino acid, ~ 12-kDa chemokine expressed in macrophages, astrocytes, endothelial cells, and epithelial cells. *CXCL9* expression is regulated by interferon (IFN)-γ response and hence is largely dependent on IFN-γ^1^. C–X–C motif chemokine receptor 3 (CXCR3), the receptor for CXCL9, is expressed on T cell subsets, natural killer cells, innate lymphoid cells, macrophages, and B-cells, all of which are related to type 1 (T1) immune reactions. IFNs can also induce the expression of CXCL10 and CXCL11, both of which share the same receptor and act in a similar manner to CXCL9.

An in vivo study using *Cxcr3*‐knockout (KO) mice indicated significant involvement of the T1 chemokine pathway in eosinophilic inflammation and airway hyperresponsiveness in an ovalbumin-induced airway inflammation model^2^. Additionally, in a cigarette-smoke-induced lung-inflammation model, *Cxcr3*-KO mice showed less lung inflammation characterised by a reduced number of CD8^+^ T cells and lower levels of IFN-γ and CXCR3 ligands (i.e., CXCL10)^3^.

Serum CXCL9 levels are reportedly elevated in some patients with asthma, interstitial lung diseases (ILDs), contact dermatitis, severe drug eruption, autoimmune diseases, and viral infections^4–11^. Patients with COVID-19 infection with high levels of serum CXCL9 develop severe conditions, such as acute respiratory distress syndrome (ARDS)^12,13^. Furthermore, serum CXCL9 levels are elevated in Stevens-Johnson/toxic epidermal necrolysis, which is caused by a type IV hypersensitivity reaction in severe drug eruptions. In contrast, the serum levels of these chemokines are not remarkably elevated in patients with drug-induced hypersensitivity syndrome, which is characterised by eosinophilic inflammation and elevated levels of T2 chemokines in the thymus and activation-regulated chemokines in the blood^7^. Additionally, immuno-suppressive or steroid treatment is beneficial for patients with ILD who have elevated serum CXCL9 levels before treatment^5^. In our previous study, we demonstrated significantly higher serum CXCL9 concentrations in patients with asthma compared to healthy controls and found an association between higher CXCL9 levels and acute exacerbation or eosinophilic asthma^4^. These results suggest that T1 inflammation is an important aspect of the inflammatory pathophysiology of patients with acute and chronic diseases.

Assessment of T1 inflammation in daily clinical practice requires a clinically reliable immunoassay. The Sysmex HISCL is an automated chemiluminescence immunoassay platform capable of detecting analyte concentrations within 17 min in 20 µL human serum or plasma samples. In the present study, we describe the development of the CXCL9 immunoassay, a new ‘research use only’ immunoassay for determining serum and plasma CXCL9 levels. We report the analytical performance of the assay in terms of precision, sensitivity, linearity, cross-reactivity, and endogenous and drug interferences, as well as the distributions of serum CXCL9 levels in samples from healthy individuals and afflicted patients. Additionally, we identified an association of several pathophysiological parameters with CXCL9, thereby characterising CXCL9 as a biomarker of T1 inflammation.

## Results

### Assay performance

Regarding precision, two levels of control (one pooled human serum and one pooled human plasma panel) were evaluated. The control concentrations were selected according to the range of healthy and unhealthy individuals. Overall, the 5-day total precision measured as percentage CV (%CV) was < 7% across the two controls, one plasma-based panel, and one serum-based panel (Table 1). The LoB, LoD, and LoQ using the HISCL 800 system were 0.22, 0.85, and 2.20 pg/mL, respectively, whereas those using the HISCL 5000 systems were 0.11, 0.32, and 1.04 pg/mL, respectively. The LoQ determined over a concentration range of 0.38 to 30 pg/mL is shown in Supplementary Fig. S1.

**Table 1:** Evaluation of the 5-day assay precision.

	Level	N	Mean CXCL9	%CV (SD)				
				Within	Between	Between	Between	Overall
			Concentration (pg/mL)	Run	Run	Day	Instrument	
HISCL-5000	High-concentration control	40	73.9	1.9 (1.4)	4.4 (3.2)	3.9 (2.9)	4.4 (3.1)	4.5 (3.4)
	Low-concentration control	40	13.1	1.9 (0.3)	3.9 (0.5)	3.4 (0.4)	0.9 (0.1)	4.2 (0.5)
	Human serum panel	40	85.6	1.4 (1.2)	3.0 (2.6)	2.7 (2.3)	3.3 (2.8)	3.3 (2.8)
	Human plasma panel	40	26.6	2.4 (0.6)	3.3 (0.9)	2.5 (0.7)	5.8 (1.5)	4.0 (1.1)
HISCL-800	High-concentration control	40	75.0	2.3 (1.7)	6.1 (4.6)	5.5 (4.2)	3.3 (2.4)	6.2 (4.7)
	Low-concentration control	40	13.6	2.1 (0.3)	5.8 (0.8)	5.2 (0.7)	2.7 (0.4)	5.9 (0.8)
	Human serum panel	40	86.1	2.8 (2.4)	3.0 (2.5)	2.7 (2.3)	1.1 (1.0)	3.9 (3.3)
	Human plasma panel	40	26.4	2.6 (0.7)	3.5 (0.9)	3.0 (0.8)	1.2 (0.3)	4.2 (1.1)

Two controls and one normal human serum panel were evaluated in four replicates. Twice daily with a ≥ 2-h gap between runs. Panels were evaluated for 5 days on two different HISCL 5000 and HISCL 800 instruments according to CLSI and EP05-A2 guidelines.

**Figure 1:**
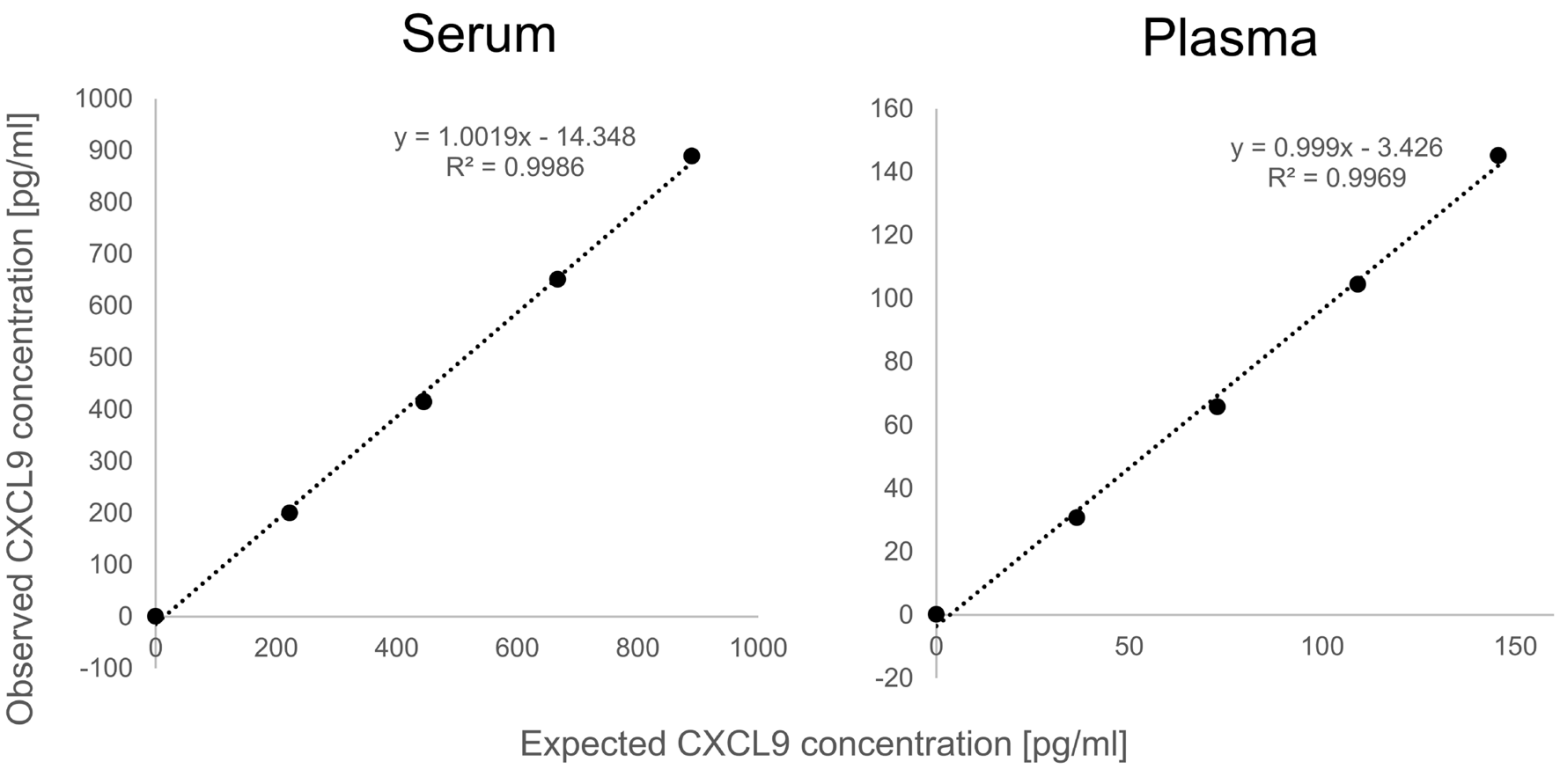
Dilution linearity. Linear regression analysis (*n* = 3) of the HISCL CXCL9 immunoassay using serum and plasma samples. R^2^, coefficient of determination.

In the specimen dilution analysis, the evaluated dynamic range was selected based on the distribution of CXCL9 levels in the plasma or serum samples. Specimen dilution analysis yielded linear results across the dynamic range of the assay. The average correlations between the observed and expected values of serum and plasma samples were 0.998 and 0.997, respectively (Fig. 1). We observed no systematic biases in the residual plots (data not shown), and the overall mean (range) spike recovery for plasma and serum samples was within 20% (Table 2).

**Table 2:** Spike-recovery test.

Spiked CXCL9 (pg/mL)	Plasma [mean % (range)]		Serum [mean % (range)]	
307	94	(89–100)	116 (113–120)	
153	91	(89–98)	107 (105–113)	
30	82	(82–85)	111 (105–117)	

Spike-recovery assays were used to determine the accuracy of the HISCL CXCL9 assay. Data show the amounts of CXCL9 measured as percent recovery of the original spiked concentration in ten individual serum or plasma samples.

**Figure 2:**
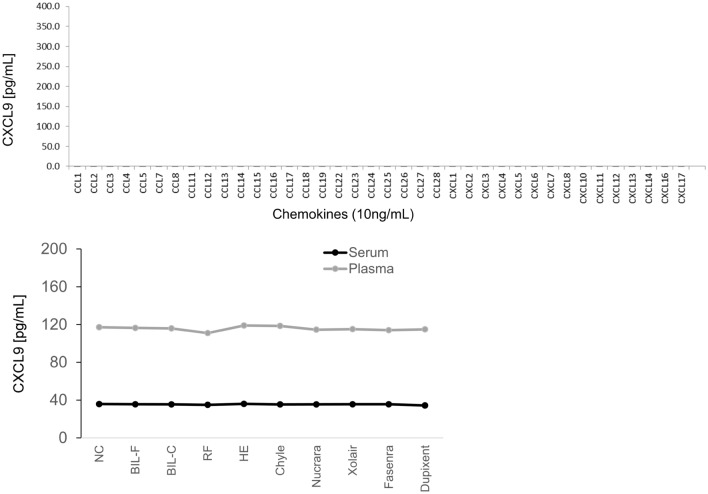
Cross-reactivity with other chemokines, compounds, and drugs. We observed no cross-reactivity with structurally similar chemokines (10 ng/mL; top). No interference was observed between CXCL9 and haemoglobin (46.0 g/L), conjugated bilirubin (BIL-F; 3450 µmol/L), unconjugated bilirubin (BIL-C; 3540 µmol/L), chyle (17,700 FTU), purified RF (500 IU/mL), and 100 μg/mL of various drugs (bottom).

In addition, we did not observe any cross-reactivity with other C–C- and C–X–C-motif chemokines (Fig. 2) or interference with bilirubin, haemoglobin, chyle, rheumatoid factor (RF), or various drugs in the serum and plasma samples (Fig. 2). Interference levels revealed a mean percentage difference of ≤ 10% between CXCL9 concentrations in the presence and absence of potential interferents. Serum CXCL9 levels did not correlate with human anti-mouse antibody (HAMA) or RF values (Supplementary Fig. S2).

This reagent will be used for several clinical studies using frozen retrospective samples. Therefore, the freeze–thaw impact for the initial values was considered the most important stability test for sample stability. Sample-stability tests showed a > 10% change in measured values before freezing and after two freeze–thaw cycles in a range of frequently observed values in samples (Supplementary Fig. S3).

**Figure 3:**
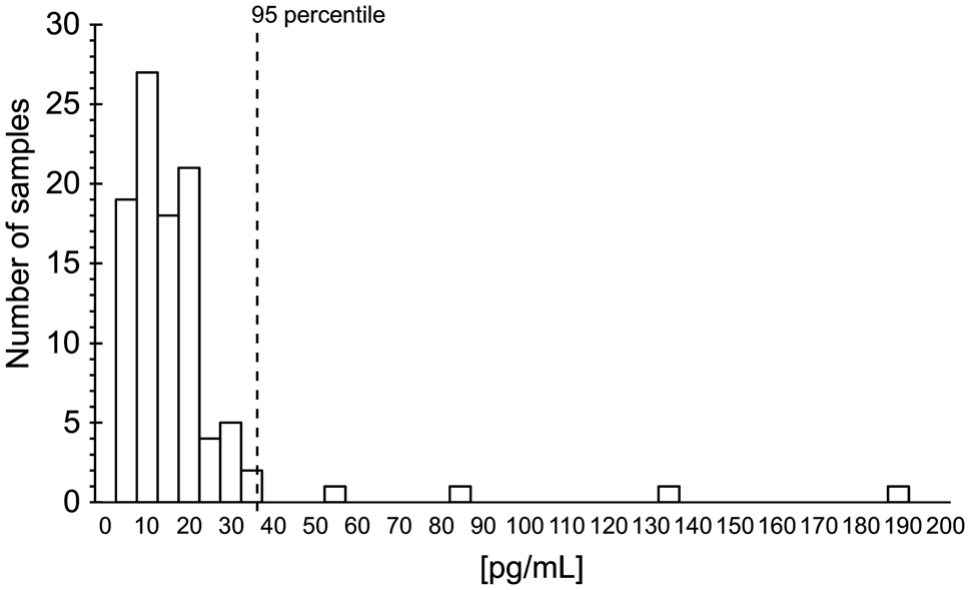
Frequency distribution of serum CXCL9 levels in healthy individuals. Results are shown as individual numbers of CXCL9 levels (open bars). The dashed line indicates the 95 percentile: 39.0 pg/mL.

Additionally, the 95 percentiles in 100 serum samples from HCs (age range 20–70 years) was 39.0 pg/mL (Fig. 3). Moreover, we observed significant differences in serum CXCL9 levels among age groups (Supplementary Fig. S4).

### Association of assay-specific CXCL9 thresholds with respiratory disease and other inflammatory cytokines

The median age of healthy control was 45.8 years; 48.7% of the individuals were male. The median age of patients with COVID-19 was 59 years; 57% of patients were male, 21.8% received oxygen therapy, 22.8% were treated by mechanical ventilation, and 49.1% suffered from ARDS. The median age of chronic bird-related hypersensitivity pneumonitis (HP) was 64 years; 54.1% of patients were male. Twenty-five patients were treated with a corticosteroid and immunosuppressants, and 20 patients were only treated with a corticosteroid. Seventy-four male and 120 female patients with asthma were recruited (median age 59.7). The median respiratory function was indicated as the forced expiratory volume (FEV)1% 71.4 (62.2–79.2). Ninety-four patients presented with atopic asthma (48%), and 13, 59, 74, and 47 patients were on medication in JGL2015 steps I, II, III, and IV, respectively. Eighty-four patients diagnosed with ILD were recruited retrospectively. Thirty-two patients were categorised as having interstitial pneumonia with autoimmune features (IPAF), and 11 patients were categorised as having collagen vascular disease-associated ILD (CVD; Supplementary Table S-1).

**Figure 4:**
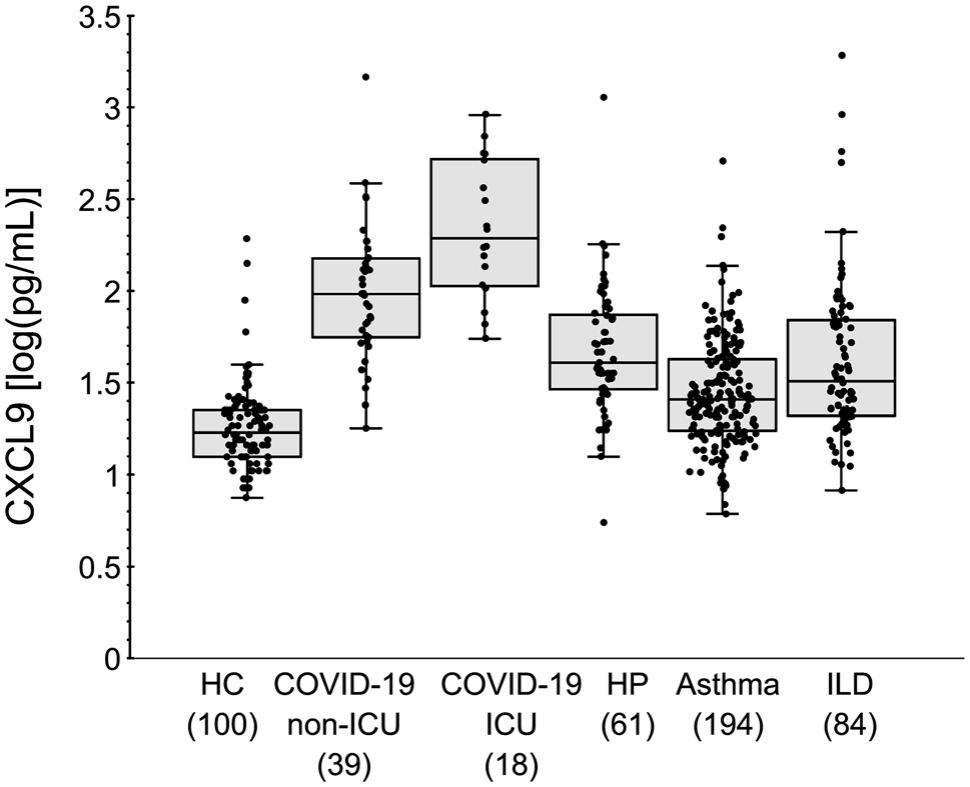
Distribution of serum CXCL9 levels. Levels of CXCL9 in HCs, COVID-19 patients not admitted to the ICU (Non-ICU) and those admitted to the ICU (ICU), and patients with asthma, ILDs, and HP, respectively. Results are shown as individual data points (circles) with medians (bars) and interquartile ranges (box). Whiskers extend to the minimum and maximum values excluding outliers.

**Figure 5:**
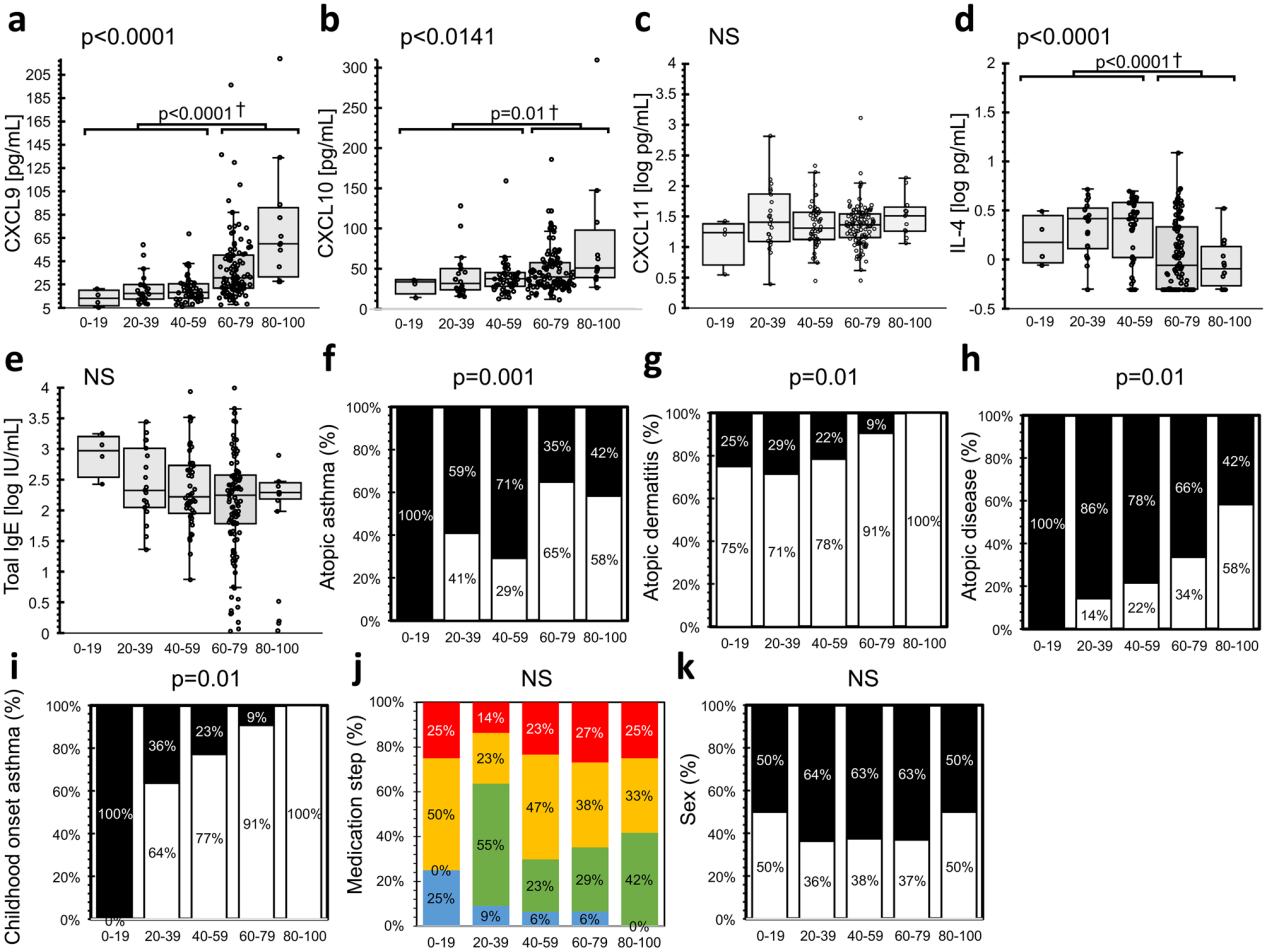
Age-related differences in CXCL9 levels in patients with asthma. Distributions of serum (**a**) CXCL9, (**b**) CXCL10, (**c**) CXCL11, (**d**) IL-4, and (**e**) total IgE levels according to age, which is indicated on the x-axis. Results are shown as individual data points with medians (bars) and interquartile ranges (box). Whiskers extend to the minimum and maximum values excluding outliers. The ratios of (**f**) atopic asthma, (**g**) comorbidity of AD (atopic dermatitis), (**h**) comorbidity of other atopic diseases (AD, atopic rhinitis, atopic conjunctivitis), (**i**) childhood-onset asthma, (**j**) medication steps in JGL2015 (Blue: I, Green: II, Yellow: III, Red: IV) and (**k**) sex are indicated. Filled bars indicate positivity or female for each condition. P-values were calculated using the Steel–Dwass test. *P < 0.05, **P < 0.01, ***P < 0.001, Mann–Whitney U (†) (**a**–**d**), and Fisher's exact tests (**f**–**k**).

We used the assay to evaluate serum samples from patients with asthma, ILDs, and HP to define the association between serum CXCL9 levels and chronic respiratory diseases (Fig. 4). We found that serum CXCL9 levels were significantly higher in patients aged > 60 years compared to HCs, (Fig. 5, Supplementary Fig. S-5). Interestingly, serum interleukin (IL)-4 levels, a Th2 cytokine, were lower in patients aged > 60 years compared to those aged < 60 (Fig. 5).

To define the relationship between CXCL9 levels and other inflammatory parameters, we compared CXCL9 levels with those of several cytokines in samples from patients with asthma. We found that CXCL9 levels significantly correlated with CXCL10 levels and moderately correlated with CCL3 levels. Additionally, the CXCL9 level weakly positively correlated with IL-6, CXCL8, CXCL11, and IL-10 levels, showing a weak negative correlation with IL-4 levels (Table 3, Supplementary Fig. S-6).

**Table 3:** Correlation of biomarkers with CXCL9 levels in patients with asthma.

	r_S_	P-value
IFN-γ	0.001	0.990
TNF-α	0.048	0.508
IL-4	− 0.186	0.009
IL-5	0.050	0.490
IL-6	0.368	< 0.0001
IL-10	0.230	0.001
IL-16	0.020	0.781
IL-17	0.013	0.860
IL-25	0.152	0.034
CCL3	0.490	< 0.0001
CCL11	0.185	0.013
CCL27	0.218	0.004
CCL17	0.044	0.545
CXCL8	0.344	< 0.0001
CXCL11	0.377	< 0.0001
CXCL10	0.626	< 0.0001

Significant correlations are indicated. The correlation coefficients were generated by Spearman’s correlation analysis. Results were considered significant at P < 0.05.

*TNF* tumour necrosis factor.

### Relationship between CXCL9 level and asthma pathophysiology

Asthma has a heterogeneous pathophysiology in patients. T2-inflammation-dependent atopic asthma with an IgE-positive profile is a well-characterised subpopulation. Atopic asthma is increased in childhood-onset asthma, whereas the onset of non-atopic asthma is predominantly observed in adult-onset asthma. In this study, we compared changes in serum levels of IL-4 and T1 chemokines and their association with CXCL9 according to the age-related presence of atopic asthma (Fig. 5). We found that serum CXCL9 levels increased with age in patients with asthma, whereas serum IL-4 and total IgE levels showed an inverse trend relative to CXCL9 levels. Additionally, the proportion of patients with atopic asthma decreased with age. Therefore, our results indicate that patients aged > 60 years have higher levels of CXCL9 and CXCL10. In particular, CXCL9 levels in 60–79 years old patients were significantly higher than in patients of 40–59 years, despite comparable treatment steps and gender ratios (Fig. 5a,j,k). Moreover, complications associated with allergic diseases, including atopic dermatitis (AD) and allergic rhinitis (AR), also decreased with age. Furthermore, the percentage of childhood-onset asthma was only 7.5% among patients aged > 60 years, with most having higher CXCL9 levels compared to HCs.

Multiple regression analysis with age as the dependent variable and CXCL9, IL-4, sex, treatment steps, and atopic asthma as explanatory variables revealed that CXCL9 concentrations, IL-4, and atopic asthma were significantly related to age (Supplementary Tables S-2, S-3).

## Discussion

In this study, we demonstrated that the LoQ of the assay was 2.2 pg/mL for the HISCL 800 system and 1.04 pg/mL for the HISCL 5000 system. Notably, serum CXCL9 levels in all HCs were above this LoQ, indicating that this reagent is sufficiently sensitive for measuring serum samples. This LoQ is lower than that of the current commercial immune ELISA system^14^. According to a previous report, the CXCL9 level in bronchoalveolar lavage fluid collected from patients with ILD was > 1 pg/mL, with some samples below the quantification range^5^. Although we did not evaluate the upper limit of quantitation in this study, the result of dilution linearity test suggests the dynamic range of our assay is covered expected ranges of CXCL9 that is observed in clinical samples (Fig. 4).

This CXCL9 assay did not exhibit cross-reactivity with chemokines having high structural similarity to CXCL9. Further, the assay sensitivity was unaffected in the presence of potential interferents in serum and plasma samples. Additionally, the two-step immunoassay protocol and use of the F(ab′)2 mAbs demonstrated good specificity. These results suggested that this reagent can be used to accurately analyse CXCL9 levels in human blood samples.

CXCR3 is a marker of T helper 1 (Th1) cells and an important component involved in the migration and functional ability in T1-mediated inflammation^15^. Serum CXCL9 levels in asthma patients are positively correlated with CXCL10 and CXCL11 levels, which are also CXCR3 ligands^4,11^. Additionally, levels of CXCL9 correlate with those of CCL3, which binds to the C–C motif chemokine receptor 5 present in Th1 cells^16^. In the present study, we found a negative correlation between CXCL9 and IL-4 levels.

Serum CXCL9 levels were < 39.0 pg/mL for HCs (Fig. 3); however, CXCL9 levels differed significantly according to age. A previous report indicated that CXCL9 levels increase with age, and our findings are consistent with this phenomenon^17^. Although minimal age-related variations were observed among HCs (Supplementary Fig. S4), the CXCL9 level was higher in HCs aged > 60 years, with values reaching 20.5 pg/mL (interquartile range 18.0–22.3 pg/mL). However, CXCL9 levels in patients with asthma and ILDs were higher than the levels in normal samples, and the difference among the age classes in the healthy subjects was extremely small compared to the disease condition (Supplementary Fig. S4). Patients with COVID-19 had elevated CXCL9 levels, which exceeded the threshold observed in HCs, even in mild cases, and were further elevated in severe cases. These results suggest that strong T1 inflammation is induced during the viral response in the acute phase of the disease.

In patients with chronic diseases, such as ILD and asthma, we found that CXCL9 levels were lower than those observed in samples from patients infected with COVID-19; however, some patients had CXCL9 levels exceeding the upper limit of the CXCL9 level in HCs. In a previous study, Kameda et al.^5^ suggested that recovery of forced vital capacity following immunosuppressive therapy was related to CXCL9 levels before treatment. Interestingly, in the present study, CXCL9 levels in therapy responders were higher than in HCs.

In patients with asthma, CXCL9 levels negatively correlated with IL-4 levels. Patients aged > 60 years had higher proportions of CXCL9 levels compared to HCs, (Fig. 5 Supplementary Fig. S-5). Earlier, the pathogenesis of asthma was classically thought to be driven by a T2 immune response; however, it is now considered a heterogeneous and complex disease. Th2-high asthma, in particular, is a condition more akin to an atopic and allergic phenotype associated with childhood-onset and responds well to classical steroid therapy, whereas Th2-low asthma is less akin to an atopic condition and is reflective of adult-onset and responds poorly to inhaled steroid therapy^17–21^. Previous studies undermine the contribution of T1-inflammatory pathophysiology in Th2-low asthma^4,22,23^. In the present study, serum levels of IL-4 and total IgE and the ratio of atopic asthma tended to decrease with age. Moreover, the ratio of childhood-onset asthma and complications of atopic diseases, such as AD and AR, also decreased in patients > 60 years of age. The multiple regression models, including sex, atopic asthma, and medication steps, also supported age-related CXCL9 levels induction, IL-4 levels, and the atopic asthma ratio reduction (Supplementary Tables S-2, S-3). Furthermore, in a comparison between 40–59 and 60–70 years old patients, CXCL9 levels were significantly higher in 60–70 years old patients, despite the sex ratio and medication condition being comparable in both populations. Therefore, significant induction of CXCL9 levels in patients > 60 years of age is not likely caused by the bias of sex or medication condition. Furthermore, these results suggest that most elderly asthmatics present an inflammatory pathology biased toward Th2-low asthmatic condition in this cohort; a finding consistent with those in previous reports^18–20,24^. The response of this population to treatments targeting T2 inflammation is of great interest.

This study has some limitations. To evaluate analytical performance, we thawed and used frozen samples, and the number of healthy specimens was insufficient to accurately evaluate the distribution of CXCL9 in the healthy population. Therefore, future clinical studies should focus on including larger sample sizes and fresh plasma samples. The existence and definition of non-T2 asthma, which comprises a range of asthma subtypes, including neutrophilic, mixed granulocytic, T1 high or Paucigranulocytic asthma^25^, is still uncertain. Therefore, it is difficult to evaluate the diagnostic performance of CXCL9. However, present data suggest a relationship between serum CXCL9 levels and respiratory inflammation, whereas the opposite trend is observed in patients with Th2 asthma.

In this study, we confirmed that the CXCL9 reagent used in an automatic immunoassay system demonstrated sufficient analytical performance for assessing the pathophysiology of inflammation regardless of the patient’s disease status (chronic or acute). Given the evidence that CXCL9 levels in inflammatory diseases are higher than in healthy subjects, our findings imply a possible association of CXCL9 with disease phenotypes.

## Methods

### Patients

This study was approved by the research and ethics committees of the National Hospital Organization (H27-NHO-04), Nara Medical University (1753), Tokyo Medical and Dental University (#G2018-003 and #G2018-004), Kobe City Medical Centre General Hospital (20041/200636), and Sysmex Corporation. All study procedures were performed in accordance with the Declaration of Helsinki. Written informed consent was obtained from all patients, or in adherence with the Ethical Guidelines for Medical and Health Research Involving Human Subjects, information on the study implementation was made public to ensure that the subjects had the opportunity to withdraw their consent at any time by publishing on the website. Therefore, written informed consent from the enrolled patients was waived by the ethics committee.

Blood donors at the Japanese Red Cross Society without subjective symptoms of infection and allergy diseases were recruited as healthy controls (HCs; average age 45.8 years; range 20–69 years; 37 males and 39 females). A group of 57 patients with COVID-19 (average age 59 years; range 45–72 years; 33 males and 57 females) diagnosed using a SARS-CoV-2-specific polymerase chain reaction test were included. Of these, 21.8% received oxygen therapy, 22.8% were treated by mechanical ventilation, and 49.1% suffered from ARDS. Sixty-one patients diagnosed with chronic bird-related hypersensitivity pneumonitis (HP) were recruited retrospectively. Patients with recurrent HP experienced repeated acute episodes of mild exertional dyspnoea, cough, and low-grade fever, whereas those with insidious HP experienced chronic, slowly progressing respiratory disease in the absence of acute episodes. Acute HP cases (approximately 70–80%) were summer-type HP cases caused by *Tricosporon*, whereas approximately 50% of chronic HP cases were bird-related. Seventy-four male and 120 female patients with asthma were recruited (median [interquartile range] 59.7 [49–70.3] years; *n* = 194). The median respiratory function was indicated as the forced expiratory volume (FEV)1% 71.4 (62.2–79.2), with 32 patients having an FEV1% < 70%. The median serum IgE level was 194 (range 79.9–501.5; *n* = 164). Ninety-four patients presented with atopic asthma (48%), and 13, 59, 74, and 47 patients were on medication in JGL2015 steps I, II, III, and IV, respectively. Eighty-four patients diagnosed with ILD were recruited retrospectively. Thirty-two patients were categorised as having IPAF, and 11 patients were categorised as having collagen vascular disease-associated ILD (CVD; Supplementary Table S-1).

Un-linkable anonymised serum and plasma samples for stability tests were provided from MEDILYS Laborgesellschaft mbH of Asklepios Klinik Altona.

### Assay development

The employed CXCL9 assay is a fully automated immunoassay operated on a HISCL-5000 or 800 system (Sysmex, Osaka, Japan). The assay has a total turnaround time of 17 min and requires serum samples for testing. The assay employs two newly developed sheep monoclonal antibodies (mAbs) that target different CXCL9 epitopes in a two-step sandwich immunoassay configuration.

Antigens in serum react with the biotin-labelled F(ab′)2 sheep mAb in the R1 reagent to form antigen–antibody complexes, followed by the addition of an R2 reagent containing streptavidin-coated magnetic beads, resulting in a strong reaction between biotin and streptavidin that immobilises the antigen on the magnetic beads. After washing with HISCL washing solution (bound/free separation), alkaline phosphatase (ALP)-labelled F(ab′)2-sheep mAb in the R3 reagent is added to promote coupling of ALP with the antigen of the immunocomplex on the magnetic beads via an antigen–antibody reaction.

After the removal of unreacted ALP-labelled antibodies, the magnetic beads are dispersed in the R4 reagent, after which the R5 reagent (CDP-*Star*) is added to generate luminescence. The intensity is then quantified, and a calibration curve is generated to determine the CXCL9 concentration (Supplementary Fig. S-7).

Six calibration points were set to range from 0 to 4000 pg/mL in a fivefold dilution series to cover the clinical sample range.

### Performance characterisation

Raw data related to analytical performance evaluation and catalogue numbers, lot numbers of specimens, and antigens have been added as supplement data.

#### Limits of blank, detection, and quantitation (LoB, LoD, and LoQ, respectively)

LoB, LoD, and LoQ were determined according to the Clinical and Laboratory Standards Institute (CLSI) document EP17-A2. The LoB was determined over 3 days (lots A and B; one instrument, eight samples, five runs daily for 3 days; *n* = 30/sample). Analyte-free sample buffer [phosphate-buffered saline (pH 7.4) and 1% bovine serum albumin) was used to assess the LoB. To assess the LoD, CXCL9 recombinant protein was diluted with sample buffer (0.45–3.4 pg/mL), and samples were analysed using one instrument (lots A and B; one run daily for 3 days; *n* = 30/sample). Whereas to determine the LoQ, CXCL9 recombinant protein (R&D systems, 392-MG, lot ABU1614052) was diluted with sample buffer (0.72–13.8 pg/mL), and samples and dilutions were tested over 3 days (1 run/day) on one instrument (lots A and B; *n* = 30/sample). The LoQ was estimated as the concentration of a 20% coefficient of variance (CV).

#### Precision testing

The assay precision was evaluated according to CLSI EP05-A2 guidelines. However, one exception was that replicates were evaluated over 5 days instead of 20 days. Two levels of control (one pooled human serum and one pooled human plasma panel) were evaluated in four replicates twice daily with a ≥ 2-h gap between runs. Panels were run for 5 days on two different HISCL 5000 or HISCL 800 instruments. For controls, recombinant CXCL9 at concentrations (R&D systems, DY392 Part No 840343, lot 1294247) of 13.1 pg/mL (low-concentration control) and 73.9 pg/mL (high-concentration control) were added into an artificial sample buffer. The human serum panel represented native or endogenous CXCL9 samples.

#### Dilution linearity and spike recovery

Dilution linearity was assessed according to CLSI guideline EP06-A. A sample of human serum with a high CXCL9 concentration was serially diluted with human serum lacking CXCL9 using magnetic microparticles coated with an anti-CXCL9 polyclonal antibody (biotin-labelled anti-CXCL9 goat IgG; R&D Systems, Minneapolis, MN, USA). The expected and observed concentrations of CXCL9 were analysed using regression analysis. Spike recovery was assessed by adding 30, 153, and 307 pg/mL recombinant CXCL9 (R&D systems, DY392 Part No 840343, lot 1294247) to ten serum and plasma samples.

#### Interferents

The potential for interference by haemoglobin (46.0 g/L), conjugated bilirubin (BIL-C; 3540 μmol/L), unconjugated bilirubin (BIL-F; 3450 μmol/L), chyle (17,700 FTU), and purified rheumatoid factor (RF; 500 IU/mL) was tested using Interference Check A Plus and Interference Check RF (Sysmex).

Additionally, we conducted experiments in triplicate to evaluate the potential interference of various drugs [including Nucara (100 μg/mL), Xorair (100 μg/mL), Fasenra (100 μg/mL), and Dupixent (100 μg/mL)], to obtain a mean percentage difference of ≤ 10% between CXCL9 concentrations in the presence and absence of interference.

#### Cross-reactivity

Cross-reactivity was observed in the presence of 10 ng/mL of C–C motif chemokine ligand, including (CCL)1, CCL2, CCL3, CCL4, CCL5, CCL7, CCL8, CCL11, CCL12, CCL13, CCL14, CCL15, CCL16, CCL17, CCL18, CCL19, CCL22, CCL23, CCL24, CCL25, CCL26, CCL27, CCL28, CXCL1, CXCL2, CXCL3, CXCL4, CXCL5, CXCL6, CXCL7, CXCL8, CXCL10, CXCL11, CXCL12, CXCL13, CXCL14, CXCL16, and CXCL17 (R&D Systems).

### Cytokine and chemokine measurements by enzyme-linked immunosorbent assay (ELISA)

All cytokines and chemokines were measured by ELISA, as described previously^22^.

### Statistical analysis

Statistical analysis was performed using Microsoft Excel (Microsoft Corp., Redmond, WA, USA) and R (http://www.r-project.org/). All values were log-transformed, outliers were excluded using the Smirnov–Grubbs test, and the standard deviation (SD) was calculated. P-values were calculated using the Kruskal–Wallis (KW) test and Fisher’s exact test, and results were considered statistically significant at P < 0.05.

## Supplementary Information

Supplementary Information 1.

Supplementary Information 2.

## Data Availability

The datasets generated and/or analysed during the current study are not publicly available due to regulation of clinical studies in Japan but are available from the corresponding author upon reasonable request. The data relating to analytical performance are included in the Supplementary Information files.
