# Supplementary material for: Development of a new HISCL automated CXCL9 immunoassay
Source: Sci Rep. 2023 Apr 1;13:5342. doi: 10.1038/s41598-023-32513-8 (PMC10066986; doi:10.1038/s41598-023-32513-8)
Supplement: Supplementary file 2 — Supplementary Information 2. [file 41598_2023_32513_MOESM2_ESM.pdf]

Supplementary information

Development of a new HISCL automated CXCL9 immunoassay

Takehiro Hasegawa, Maho Yoshida, Shunsuke Watanabe, Takami Kondo, Hideo Asada, Atsushi Nakagawa, Keisuke Tomii, Masami Kameda, Mitsuo Otsuka, Koji Kuronuma, Hirofumi Chiba, Shinji Katayanagi, Yasunari Miyazaki, Akio Mori

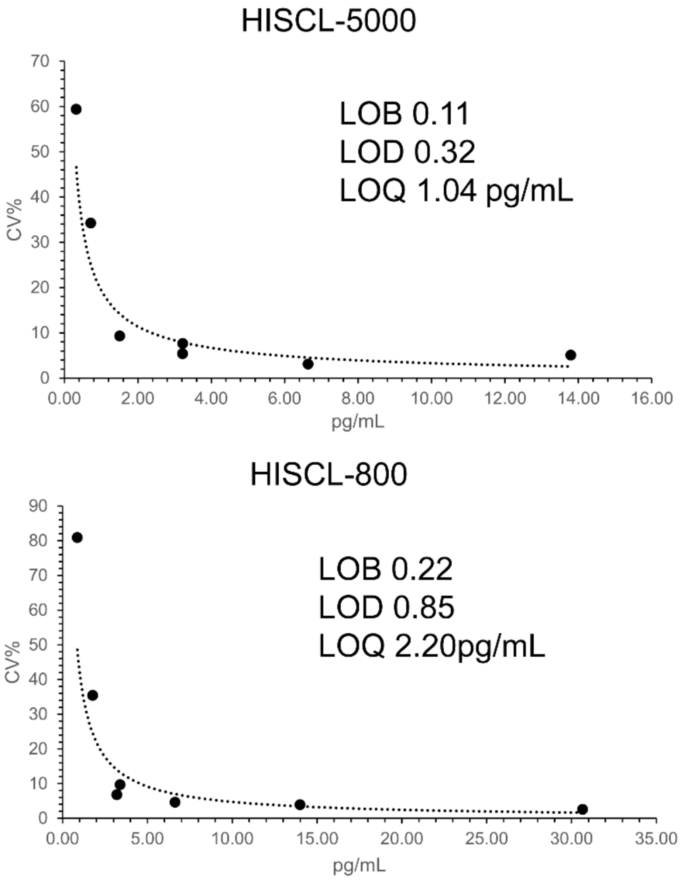

Figure S1. Lower LoQ of the assay. LoQ was assessed by measuring seven samples (0.38–30 pg/mL) on two different HISCL instruments and estimated as the concentration with an intermediate precision of 20% coefficient of variation.

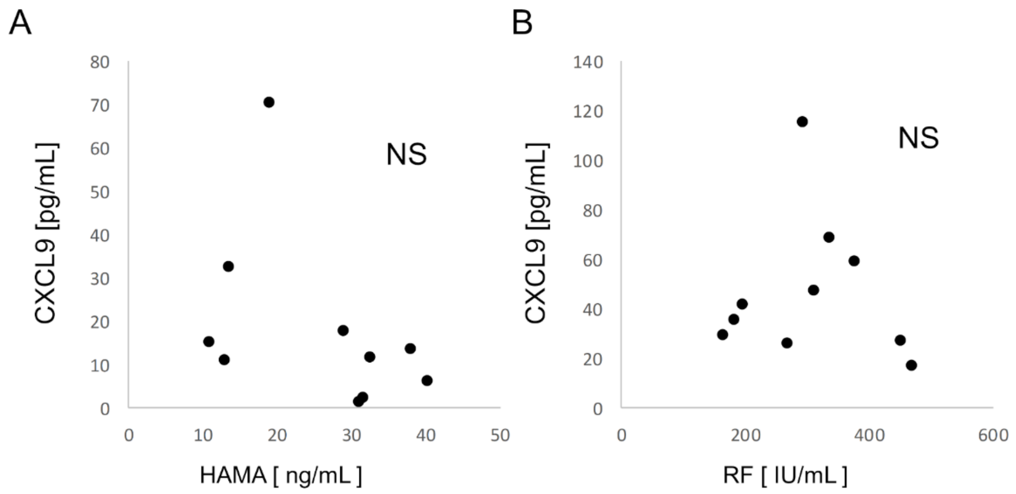

Figure S2. Correlations of levels of HAMA and RF with CXCL9 levels. Comparisons of serum CXCL9 levels with interference by (**a**) human anti-mouse antibody (HAMA) and (**b**) rheumatoid factor (RF). Correlations were analysed using Spearman's rank correlation test. NS: non-significant

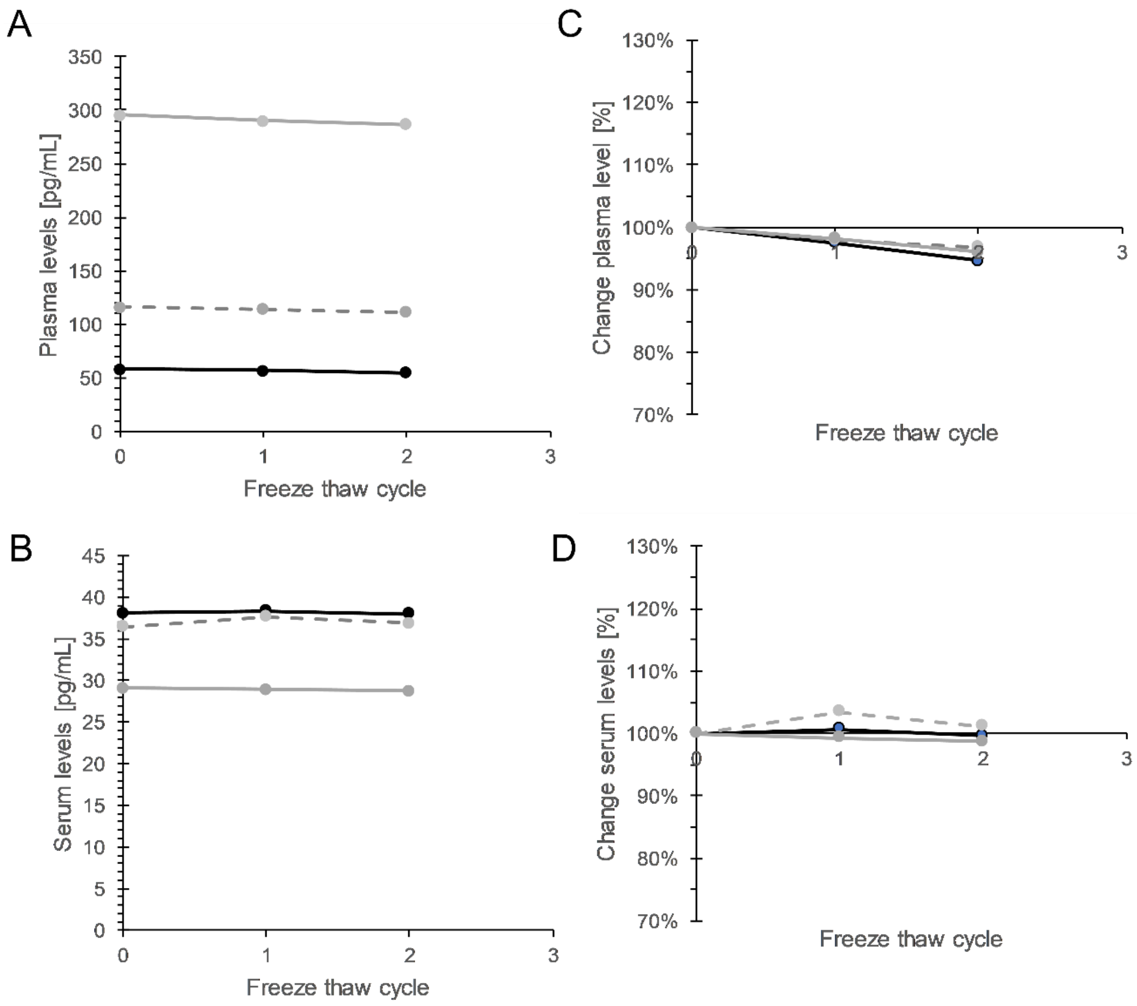

Figure S3. Effects of freeze-thaw cycles on plasma and serum samples. Each line indicates the same sample in each graph. (**a, b**) Measured levels in plasma and serum are indicated, respectively. (**c, d**) Percent changes in the measured plasma and serum values before freezing, respectively.

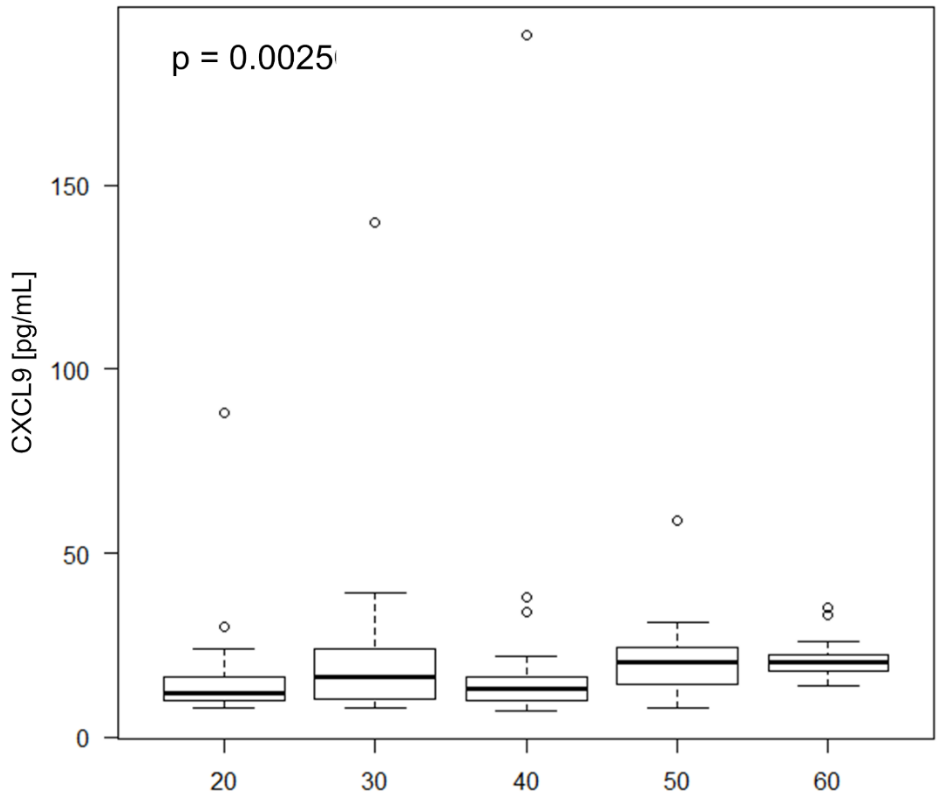

Figure S4. Age-related distribution of serum CXCL9 levels in HCs. Results are shown as individual data points with medians (bars) and interquartile ranges (box). Whiskers extend to the minimum and maximum values excluding outliers. The P-value was calculated using the Kruskal–Wallis test.

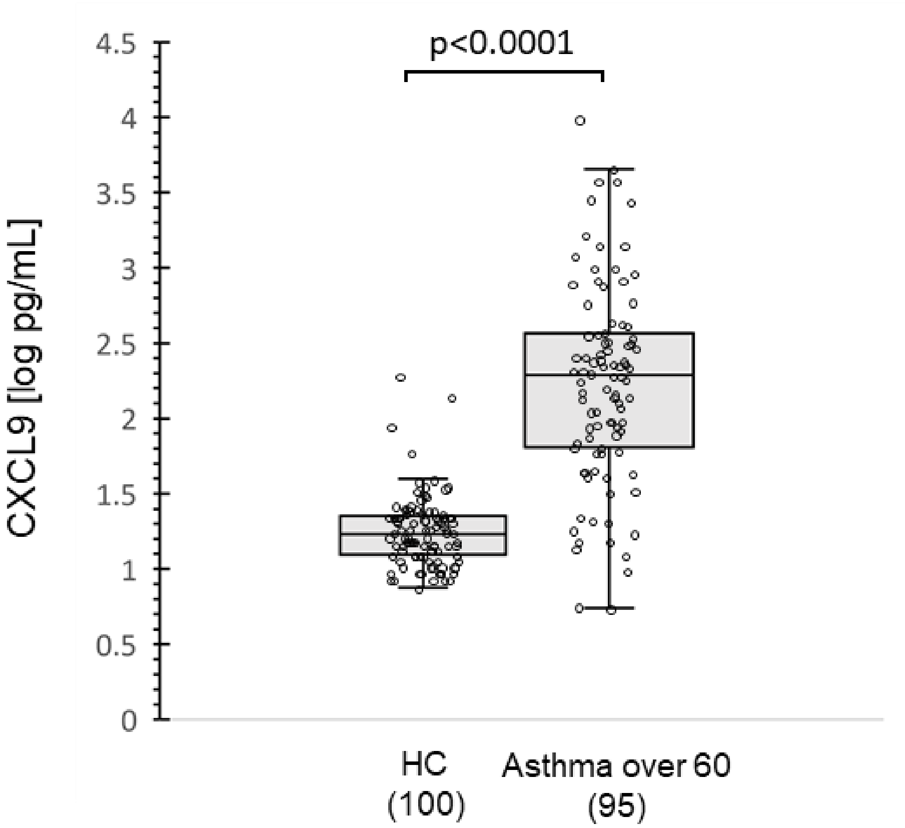

Figure S5. serum CXCL9 levels in HCs and Asthmatics over 60 years old. Results are shown as individual data points with medians (bars) and interquartile ranges (box). Whiskers extend to the minimum and maximum values excluding outliers. The P-value was calculated using the Welch's T test.

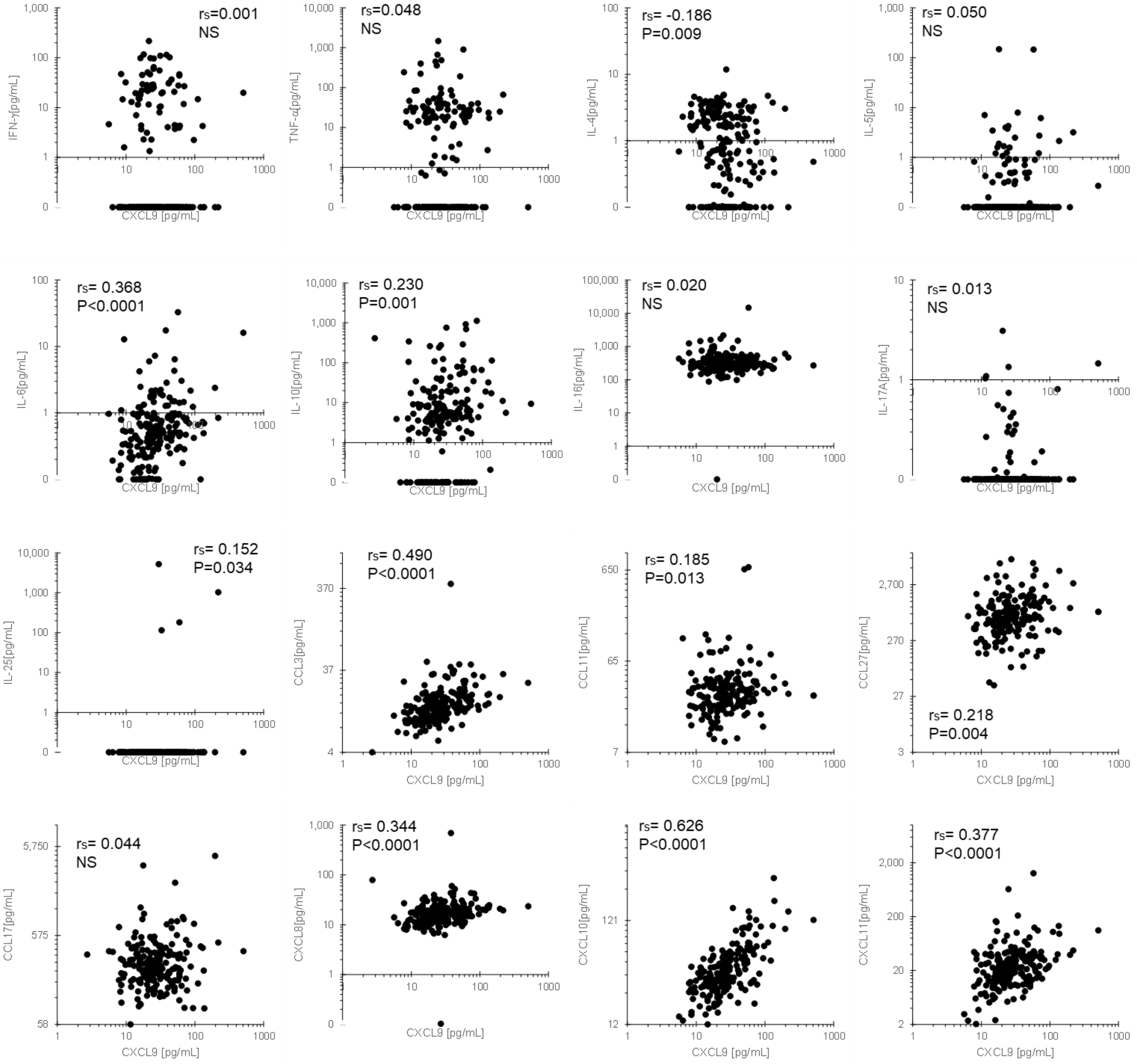

Figure S6. Correlation of biomarkers with CXCL9 levels in patients with asthma.

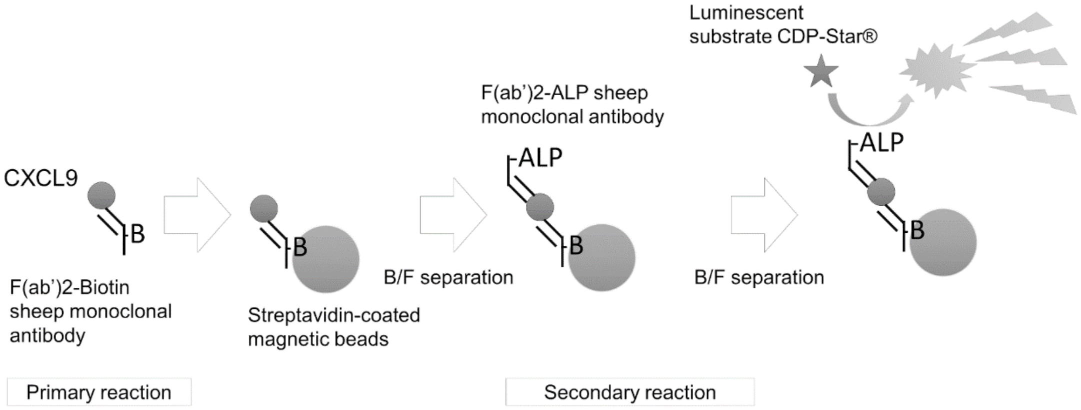

Figure S7. HISCL CXCL9 Immunoassay protocol. The sample (20 µL) is mixed with 50 µL of a biotin-labelled antibody, followed by the addition of 30 µL of streptavidin-coated magnetic beads. The antigen-antibody complex is washed, and 100 µL of ALP-labelled antibody solution is then added. After second bound/free separation, the ALP activity is detected by the substrate (CDP star).

Table S-1 Demographic and clinical data

	HC	COVID-19*	HP*	Asthma	ILD*
Sex, (F/M, n)	39(51%) / 37(48.7%)	24 (42.1%) / 33 (57.9%)	28 (45.9%) / 33 (54.1)	120 (61.9%) / 74(38.1%)	34 (40.5%) / 50 (59.5%)
Age, (Median, 1Q-3Q)	45.8 (20.5-69)	59 (45–72)	64.0 (56.5-71.0)	63 (49-70.25)	64.0 (62.0-74.0)
Childhood onset asthma, (n)	NA	NA	NA	29 (15%)	NA
Atopic asthma†, (n)	NA	NA	NA	94 (48%)	NA
Histological patterns (n)	NA	NA	UIP 11/ fNSIP 8/ cNSIP1[a]	NA	NA
HRCT patterns (n)	NA	NA	NA	NA	UIP 37/NSIP 19/Possible UIP12/ Unclassifiable 4/OP 4/ PPFE 5/Unclassifiable (NSIP) 3
Total IgE, (IU/mL, Median, 1Q-3Q)	NA	NA	NA	193.5 (79.6-501.5)[b]	NA
Serum KL-6, (IU/mL, Median, 1Q-3Q)	NA	NA	1,182 (552–1,965)	NA	886.6 (477.3–1,328.8)
Blood eosinophil (%, Median, 1Q-3Q)	NA	NA	NA	4.7 (2.3-9.7)[c]	NA
Sputum eosinophil positive, (n, %)	NA	NA	NA	106 (55%)[d]	NA
Fever (°C)	NA	38.0 (36.9–38.4)	NA	NA	NA
SpO2 (%)	NA	95 (94–98)	NA	NA	NA
FEV1%, (Median, 1Q-3Q)	NA	NA	NA	71.4 (62.2-79.2)[e]	NA
%VC, (Median, 1Q-3Q)	NA	NA	78.7 (65.4–86.2)	NA	90.6 (76.6–108.5)
%DLco, (Median, 1Q-3Q)	NA	NA	55.9 (39.6–66.2)	NA	55.7 (44.9–68.4)
Autoimmune (n)	NA	NA	NA	NA	IPAF 32/ CVD 11
Medication steps asthma, (n, %)					
I	NA	NA	NA	13 (7%)	NA
II	NA	NA	NA	59 (30%)	NA
III	NA	NA	NA	74 (38%)	NA
IV	NA	NA	NA	47 (24%)	NA
Corticosteroid and immunosuppressants	NA	NA	25 (41.0%)	NA	0 (0%)
Corticosteroid	NA	0 (0%)	20 (32.8%)	16(8%)	0 (0%)
Nasal cannula oxygen therapy	NA	12 (21.16%)	NA	NA	NA
Invasive mechanical ventilatio	NA	13 (22.8%)	NA	NA	NA
ICU	NA	18 (31.6%)	NA	NA	NA

*Samples of Hypersensitivity pneumonitis (HP) and COVID-19 are identical to those previously reported [5, 6,12]; †Atopic asthma patients have serum IgE antibody against at least one perennial airborne allergen; a: n=20; b: n=164; c: n=189; d: n=106; e: n=189; Medication steps asthma were classified according to JGL2015. HRCT: High-resolution computed tomography; KL-6: Krebs von den Lungen 6; SpO2: saturation of percutaneous oxygen; FEV1%: Forced expiratory volume in one second percentage; VC: vital capacity; DLco: diffusing capacity of the lung for carbon monoxide; ICU: intensive care unit; UIP: usual interstitial pneumonia; NSIP: Nonspecific Interstitial Pneumonia; OP: organizing pneumonia; PPFE: Pleuroparenchymal fibroelastosis; IPAF: Interstitial Pneumonia with Autoimmune Features; CVD: collagen vascular diseases associated ILD; NA: Not Applicable

Table S-2 Multiple regression analysis of predictors of CXCL9 levels

	β	Std.Error	t value	P-value	
(Intercept)	0.732	0.265	2.763	0.0063	**
Age	0.009	0.001	8.276	2.36E-14	***
Sex	-0.054	0.038	-1.424	0.156	
Step I	0.235	0.264	0.889	0.3753	
Step II	0.190	0.257	0.74	0.4605	
Step III	0.161	0.257	0.629	0.5302	
Step IV	0.177	0.258	0.687	0.4929	

Step: Medication steps asthma were classified according to JGL2015
Std.Error: Standard Error, *: $p<0.05$; **: $p<0.01$; ***: $p<0.001$

Table S-3 Multiple regression analysis of predictors of Age

	β	Std.Error	t value	P-value	
(Intercept)	27.661	13.435	2.059	0.041	*
IL-4	-10.994	2.762	-3.981	9.870E-05	***
CXCL9	26.567	3.142	8.456	8.170E-15	***
Sex	1.013	1.902	0.533	0.595	
Step I	-6.095	13.201	-0.462	0.645	
Step II	-2.721	12.822	-0.212	0.832	
Step III	-0.757	12.793	-0.059	0.953	
Step IV	-1.292	12.869	-0.100	0.920	
Atopic asthma	-7.337	1.895	-3.872	1.500E-04	***

Step: Medication steps asthma were classified according to JGL2015
Std.Error: Standard Error, *: $p<0.05$; **: $p<0.01$; ***: $p<0.001$
